# Limited Diagnostic Utility of Cortisol and Growth Hormone Levels Obtained during Hypoglycemia in Children

**DOI:** 10.1159/hrp/adaag003

**Published:** 2026-09-07

**Authors:** Merav Gil Margolis, Michal Yackobovitch-Gavan, Moshe Phillip, Liat de Vries

**Affiliations:** a The Jesse Z and Sara Lea Shafer Institute for Endocrinology and Diabetes, National Center for Childhood Diabetes, Schneider Children’s Medical Center of Israel, Petach Tikva, Israel; b Gray Faculty of Medical and Health Sciences, Tel Aviv University, Tel Aviv, Israel; c Department of Epidemiology and Preventive Medicine, School of Public Health, Gray Faculty of Medicine and Health Sciences, Tel Aviv University, Tel Aviv, Israel

**Keywords:** Adrenal insufficiency, Hypoglycemia, Critical sample, Growth hormone, Cortisol

## Abstract

**Introduction:**

Pediatric hypoglycemia has heterogeneous, age-dependent causes. Both growth hormone (GH) deficiency and adrenal insufficiency (AI) may be present with hypoglycemia; AI can lead to fatal adrenal crisis. Our objectives were to assess cortisol and GH responses to spontaneous hypoglycemia and determine the prevalence of AI (cortisol <500 nmol/L).

**Methods:**

Retrospective cohort was comprised of 133 children (1992–2022) with laboratory glucose ≤50 mg% during hypoglycemia.

**Results:**

In critical samples (CSs), cortisol <500 nmol/L was observed in 51.1% (*n* = 68) and <276 nmol/L in 23.3% (*n* = 31). AI was ultimately diagnosed in 5 patients (3.7%): four with multiple pituitary hormone deficiency and one with primary AI. Children with CS cortisol <500 nmol/L had higher median CS GH than the rest of the cohort (7.6 vs. 3.7 ng/mL, *p* < 0.001) and more frequent detectable insulin (53.3% vs. 35.6%, *p* = 0.052), with no other clinical or biochemical differences. CS cortisol correlated with fasting test nadir cortisol (*r* = 0.574, *p* < 0.001) and Synacthen-stimulated cortisol (*r* = 0.448, *p* = 0.004), but not with age (*r* = 0.026, *p* = 0.765) or CS glucose (*r* = 0.025, *p* = 0.802). CS cortisol was inversely associated with CS insulin (*r* = −0.242, *p* = 0.009). A CS GH <7.5 ng/mL was present in 56.9% of patients. CS GH showed inverse associations with age (*r* = −0.489, *p* < 0.001) and glucose (*r* = −0.332, *p* < 0.001), but not with height-SDS or weight-SDS (*r* = −0.046, *p* = 0.694; *r* = −0.021, *p* = 0.819).

**Conclusions:**

Blunted cortisol responses to spontaneous hypoglycemia in children are frequent, whereas AI is rare. In the absence of a reliable clinical discriminator, Synacthen testing is indicated. Suboptimal GH responses are also common, and further assessment should be guided by clinical judgment.

## Introduction

Hypoglycemia reflects an imbalance between supply and consumption of glucose and alternative fuels and may result from a multitude of disturbed regulatory mechanisms. Despite the expanding knowledge and use of next-generation genetic tools, the diagnosis of childhood hypoglycemia remains a challenge, with no definite diagnosis reached for a substantial number of children [1, 2]. The rate and etiology of hypoglycemia in the pediatric emergency room have been described in the literature [3, 4] and include poisoning, critical illness, as well as relatively benign diagnoses such as ketotic hypoglycemia and transient hyperinsulinism in low-birth-weight newborns.

A physiological decline in blood glucose triggers suppression of insulin secretion and activation of counter-regulatory hormones, notably cortisol and growth hormone (GH). Deficiency or insufficiency of these hormones predisposes to hypoglycemia, particularly under stress. While normative cortisol and GH responses to insulin-induced hypoglycemia during dynamic testing in children are well described, data on their responses to spontaneous hypoglycemia – often more insidious in onset – remain limited.

Recognition of hypoglycemia as a symptom of adrenal insufficiency (AI) is important to prevent treatable causes of sudden death. Affected neonates appear especially susceptible to the compromise of these counter-regulatory mechanisms, but affected older children and adults also remain at risk.

It is not uncommon to obtain a critical sample (CS) cortisol less than 500 nmol/L, but the prevalence of AI as an etiologic factor in hypoglycemia remains poorly defined. In adults, AI has been reported to account for up to 6.1% of hypoglycemia cases presenting to emergency departments [5]. Among infants and young children, hypoglycemia is a relatively common manifestation during acute illness [6]; nevertheless, the prevalence of AI as an underlying cause in this population has not been established. In the present study, we aimed to evaluate the responses of cortisol and GH to spontaneous hypoglycemia in infants and children and to assess the rate of AI or GH deficiency in these children.

## Methods

### Patients

The medical files of 325 patients with a diagnosis of hypoglycemia were reviewed. Files were retrieved according to ICD-10 diagnosis codes and from our local registry of patients with hypoglycemia to ensure complete data coverage, as the use of electronic medical files was introduced in our medical center only in 2007. Included were children aged 0–18 years who had been referred to our pediatric endocrine institute for hypoglycemia during 1992–2022. A hypoglycemic episode was defined as a serum laboratory glucose level ≤50 mg/dL associated with hypoglycemia symptoms. In asymptomatic patients, or when doubt was raised regarding the clinical presentation, patients were included only if hypoglycemia was confirmed in the laboratory for more than one episode. Excluded were patients with type 1 diabetes (*n* = 84), insufficient data (*n* = 29), and those with hypoglycemia unconfirmed by laboratory testing (*n* = 67). Also excluded were patients with hypoglycemia induced by poisoning, medications, alcohol intoxication, or critical illness (*n* = 12).

Neonates are referred to the endocrine clinic if hypoglycemic episodes do not resolve by date of discharge (at least 72 h postdelivery) and require medical and/or nutritional therapy. The same criteria were applied in our previously published cohort [2], in which neonates meeting this threshold had predominantly hyperinsulinism (48.3% transient, 36.2% persistent), supporting the clinical significance of this subgroup.

### Methodology

Patient data were collected at diagnosis and at follow-up visits. These included demographics, birth weight, anthropometric measurements, and pubertal stage according to Tanner; family history of diabetes, hypoglycemia, inborn errors of metabolism, and sudden infant death; clinical presentation; precipitating events; and biochemical and endocrine results at admission and during the work-up.

Weight standard deviation scores (Wt-SDS), BMI, BMI-SDS, and height-SDS (Ht-SDS) were calculated in accordance with the Centers for Disease Control and Prevention recommendations [7]. Recumbent length was used for children up to 3 years of age.

A serum cortisol level ≥500 nmol/L, either in a CS or after Synacthen (Novartis, NY, USA) stimulation, was considered a normal response [8]. Serum cortisol was measured using the Coat-A-Count radioimmunoassay (Diagnostic Products Corporation, CA, USA) and serum GH concentration by a chemiluminescent immunoassay using an Immulite Automated Analyser (Diagnostic Products Corporation, CA, USA).

A GH serum level <7.5 ng/mL was considered an inadequate response. Given the limited sensitivity of GH measurement in CSs [9], the decision to evaluate GH by stimulation test was at the discretion of the treating endocrinologist. Clinician-guided selection for GH stimulation reflects standard pediatric practice and ethical constraints. Electronic medical files of the entire cohort were reviewed for further hypoglycemic episodes, additional hospital visits for hypoglycemia, and endocrine and other outpatient notes or additional diagnoses documented during the period between the last visit and data collection.

### Statistical Analysis

The data were analyzed using IBM SPSS Statistics for Windows v29 (IBM Corp., Armonk, NY, USA). Data are presented as mean ± standard deviation for variables with a normal distribution, median, and interquartile range for variables with a skewed distribution, and *n* (%) for categorical variables.

Patients with CS serum cortisol levels <500 nmol/L were compared with those with cortisol levels ≥500 nmol/L. In the absence of a universally accepted cortisol cutoff for diagnosing AI during hypoglycemia in children, a CS cortisol threshold of 276 nmol/L – corresponding to the accepted normal morning cortisol level [10] – was used to further characterize patients with a low CS cortisol response. Patients with CS cortisol <276 nmol/L were compared with the remainder of the cohort. Additionally, patients with CS serum GH levels <7.5 ng/mL were compared with those with GH levels ≥7.5 ng/mL. Between-group comparisons were evaluated using an independent sample t test for normally distributed variables, the Mann-Whitney U test for variables with a skewed distribution, and Pearson’s chi-square test for categorical variables. Associations between CS serum cortisol (skewed distribution) and different clinical parameters were assessed using Spearman’s correlations.

## Results

The cohort comprised 133 patients, 79 (59%) of whom were males. Clinical characteristics are summarized in Table 1. The median age at first hypoglycemic episode was 0.88 years (interquartile range 0–2.97). Hypoglycemia occurred in 48 (36.1%) patients during the neonatal period, 22 (16.5%) during infancy, and 63 (47.4%) after 12 months of age, including 48 aged 1–6 years and 15 aged 6–18 years. Of the 48 neonates, 33 (68.8%) were born at term, 14 (29.2%) were late preterm, and 1 (2.0%) was moderate preterm. Among the 48 neonates, final diagnoses were transient hyperinsulinism (*n* = 23, 47.9%), persistent hyperinsulinism (*n* = 12, 25.0%), hormone deficiency (*n* = 6, 12.5%), systemic disease or syndrome (*n* = 4, 8.3%), ketotic hypoglycemia (*n* = 1, 2.1%), metabolic disorder (*n* = 1, 2.1%), and unknown etiology (*n* = 1, 2.1%).

**Table 1.** Clinical characteristics of the study cohort: children with spontaneous hypoglycemia

Clinical characteristics	
Age at 1st hypoglycemic episode (median, IQR), years	0.88 (0–2.97)
Male, *n* (%)	79 (59.4)
Ethnicity, *n* (%)	
Jews	125 (94)
Arabs	8 (6)
Weight for gestational age, *n* (%)	
AGA	97 (72.9)
Small for gestational age	16 (12.0)
Large for gestational age	13 (9.8)
Missing data	7 (5.3)
Wt-SDS at 1st visit, median (IQR)	−0.90 (−2.02 to 0.09)
Ht-SDS at 1st visit (mean±SD)	−0.69±1.31
Clinical presentation, *n* (%)	
Vomiting, sweating, hypothermia	4 (3)
Weakness, syncope, behavioral changes	61 (45.8)
Neonatal hypoglycemia	39 (29.3)
Seizures, tremor, jitteriness	18 (13.5)
Asymptomatic	9 (6.8)
Missing data	2 (1.5)
Hypoglycemia circumstances, *n* (%)	
Fasting	59 (44.3)
Spontaneous	26 (19.5)
Postprandial	1 (0.7)
Unknown	43 (32.3)
Missing data	4 (3.0)
CS glucose, median (IQR), mg%	39 (28–45)
Detectable insulin in CS, *n* (%)	53 (39.8)
Non-detectable insulin in CS, *n* (%)	66 (49.6)
Missing, *n* (%)	14 (10.6)
CS GH, median (IQR), ng/mL	5.6 (1.9–14.1)
CS cortisol, median (IQR), nmol/L	480 (280–807)
Fasting test peak cortisol (mean±SD), nmol/L	546±238
Missing	33
Synacthen-stimulated peak cortisol (mean±SD), nmol/L	799±290
Diagnosis, *n* (%)	
Unknown	9 (6.8)
Persistent hyperinsulinism	26 (19.5)
Transient hyperinsulinism	23 (17.3)
Ketotic hypoglycemia	36 (27.1)
Metabolic hypoglycemia	11 (8.3)
Hormone deficiency/insensitivity	13 (9.8)
Systemic disease/syndrome	14 (10.5)
Insulinoma	1 (0.7)

CS, critical sample; IQR, interquartile range; AGA, appropriate for gestational age.

### Cortisol in CSs

Cortisol concentrations <500 nmol/L were observed in 68 (51.1%) CSs, and <276 nmol/L in 31 (23.3%) patients. A normal response to Synacthen stimulation or a cortisol level >500 nmol/L on a single sample on a different occasion was documented in 63 (92.6%) of the 68 cases with CS cortisol <500 nmol/L.

AI was diagnosed in 5 patients (3.7%), as shown in Table 2: one with primary AI secondary to Kearns-Sayre syndrome and four with multiple pituitary hormone deficiency (MPHD). Three of the 4 MPHD patients presented with hypoglycemia on the first day after birth. The fourth had prolonged jaundice leading to a diagnosis of central hypothyroidism. A random basal glucose sampling during a glucagon test performed as part of the evaluation of GH reserve was 49 mg/dL. Basal and Synacthen-stimulated cortisol levels at 5 weeks of age were 86 and 574 nmol/L, respectively. Reevaluation with a Synacthen test at 5 months of age showed a peak cortisol of 237 nmol/L.

**Table 2.** Clinical characteristics of children with AI presenting with hypoglycemia

​	Age at 1st hypoglycemia	Gestational age, weeks	Birth weight, kg	Clinical presentation	CS glucose, mg/dL	Insulin, µ/mL	TSH/FT4 mIU/L/pmol/L	Peak stimulated GH, ng/mL	CS cortisol, nmol/L	Synacthen-stimulated cortisol, nmol/L	Diagnosis
1	≤24 h postnatal	41	3.8	Neonatal asymptomatic hypoglycemia	44	0.7	3.83/9.9	1.59	29	435	MPHD d/t FOXA2 mutation
2	≤24 h postnatal	41	3.7	Neonatal jitteriness	36	0.6	1.12/11.46	2.43	57	166	MPHD
3	5 weeks	41	3.2	Prolonged jaundice, asymptomatic hypoglycemia	34	0.4	1.8/1.3	0.5	74	At 5 wks – 574	MPHD
										At 5 mos – 237	
4	4 yr 11 mo	40	3.7	Adrenal crisis	22	0.4	5.2/13.9	20.6	27	301	Primary AI d/t Kearns-Sayre syndrome
5	≤24 h postnatal	40	3.6	Neonatal hypoglycemia	4	<2.0	0.22/12.6	5	<27.6	At 2 days – 469	MPHD
										At 5 days – 532	
										At 60 days − 301	

MPHD, multiple pituitary hormone deficiency; CS, critical sample; GH, growth hormone.

All 5 patients had CS cortisol levels below 100 nmol/L; however, 6 additional patients with CS <100 nmol/L demonstrated a normal cortisol response to Synacthen stimulation and did not develop AI during long-term follow-up. Three of these presented during the neonatal period and were diagnosed with congenital hyperinsulinism, transient hyperinsulinism, and prematurity-associated hypoglycemia. The other three were older children diagnosed with ketotic hypoglycemia.

### Comparison by CS Cortisol Level

Children with CS serum cortisol <500 nmol/L (*n* = 68, 51%) had a significantly lower median Wt-SDS (−1.47 [−2.37, 0.01] vs. −0.72 [−1.57, 0.35], *p* = 0.021), with no significant differences in Ht-SDS (Table 3). Hypoglycemia occurred after fasting in 58.7% of those with cortisol levels ≥500 nmol/L, compared with only 33.3% of those with cortisol levels <500 nmol/L (*p* = 0.008). There was a trend toward higher insulin detection in those with cortisol levels <500 nmol/L (53.3% vs. 35.6%, *p* = 0.052), and the median GH level in CSs from this group was significantly higher (7.6 vs. 3.7 ng/mL, *p* < 0.001).

**Table 3.** Clinical characteristics of children with spontaneous hypoglycemia stratified by CS cortisol levels (≥500 vs. <500 nmol/L)

​	Cortisol <500 (*n* = 68)	Cortisol ≥500 (*n* = 65)	*p* value
Age, median (IQR), years	0.5 (0, 3.1)	1.8 (0, 2.9)	0.221
Male gender, *n* (%)	42 (61.8)	37 (56.9)	0.570
Birth weight, *n* (%)			0.557
AGA	47 (69.1)	50 (80.0)	
Small for gestational age	10 (14.7)	6 (9.2)	
Large for gestational age	6 (8.8)	7 (10.7)	
Missing	5 (7.3)	2 (3.0)	
Wt-SDS, median (IQR)	−1.47 (−2.37, 0.01)	−0.72 (−1.57, 0.35)	0.021
Ht-SDS, mean±SD	−0.72±1.39	−0.58±1.18	0.621
Hypoglycemia timing, *n* (%)			0.008
Fasting	22 (33.3)	37 (58.7)	
Postprandial	15 (22.7)	12 (18.4)	
Unknown	29 (43.9)	14 (22.2)	
CS serum glucose, median (IQR), mg/Dl	38 (27.0, 45.0)	40.5 (32.5, 45.0)	0.364
Detectable insulin in CS, *n* (%)	32/60 (53.3)	21/59 (35.6)	0.052
Missing	8/60 (13.3)	6/59 (10.2)	
CS GH, median (IQR), ng/mL	7.6 (3.2, 16.4)	3.7 (1.7, 12.6)	<0.001
Missing	10	6	
Diagnosis, *n* (%)	​	​	0.011
Unspecified	3 (4.4)	6 (9.2)	​
Persistent HH	10 (14.7)	16 (24.6)	​
Transient HH	17 (25.0)	6 (9.2)	​
Ketotic hypoglycemia	14 (20.6)	22 (33.8)	​
Metabolic disease	4 (5.9)	7 (10.8)	​
Hormone deficiency/insufficiency	10 (14.7)	3 (4.6)	​
Syndrome/systemic disease	10 (14.7)	4 (6.2)	​
Insulinoma	0	1 (1.5)	​
Fasting test peak cortisol, mean±SD, nmol/L	386.6±209.0	646.0±206.1	0.002
*N*	11	22	

HH, hyperinsulinemic hypoglycemia; CS, critical sample; IQR, interquartile range; AGA, appropriate for gestational age.

Compared with the rest of the cohort, children with CS cortisol <276 nmol/L (*n* = 31) were younger (0 [0, 1.2] vs. 1.5 [0, 3.2] years, *p* = 0.003) and had lower CS median glucose (33.5 [20.3, 39.0] vs. 40.0 [31.0, 45.0] mg/dL, *p* = 0.019). Hypoglycemia was postprandial in 31% of children with CS cortisol <276 nmol/L compared with 17% in the rest of the cohort (*p* = 0.002).

Fifty-two (39.1%) patients completed a supervised fasting test as part of the diagnostic evaluation. Hypoglycemia (glucose ≤50 mg/dL) occurred in 40 (76.9%) patients after a mean of 17.14 ± 0.7 h (range 4–36). Among these 40 patients, diagnoses were ketotic hypoglycemia (*n* = 18, 45.0%), persistent hyperinsulinism (*n* = 7, 17.5%), inborn errors of metabolism (*n* = 3, 7.5%), systemic disease or syndrome (*n* = 3, 7.5%), insulinoma (*n* = 2, 5.0%), transient hyperinsulinism (*n* = 1, 2.5%), and hormone deficiency/insufficiency (*n* = 1, 2.5%); 5 patients (12.5%) received no definitive diagnosis.

Low CS cortisol (<500 nmol/L) was more frequent in neonates than in older children (60.4% vs. 45.8%, *p* = 0.148), while low CS GH (<7.5 ng/mL) was significantly less common in neonates compared with the rest of the cohort (23.2% vs. 75%, *p* < 0.001). Age-stratified cortisol and GH outcomes are presented in Table 4.

**Table 4.** Proportion of patients with low CS cortisol and GH by age group

​	0–1 month	>1–12 months	>12 months	*p* value
Cortisol measurements, *n*	48	22	63	​
GH measurements, *n*	43	23	57	​
CS cortisol <500 nmol/L, *n* (%)	29 (60.4)	14 (63.6)	25 (39.7)	0.042
CS cortisol ≥500 nmol/L, *n* (%)	19 (39.6)	8 (36.4)	38 (60.3)	
CS GH <7.5 ng/mL, *n* (%)	10 (23.3)	13 (56.5)	47 (82.5)	<0.001
CS GH ≥7.5 ng/mL, *n* (%)	33 (76.7)	10 (43.5)	10 (17.5)	

Percentages are of total patients in that age group with available data.

GH, growth hormone; CS, critical sample.

### Correlations

CS cortisol was associated with fasting test nadir cortisol (*r*_s_ = 0.574; *p* < 0.001) and Synacthen-stimulated cortisol (*r*_s_ = 0.448; *p* = 0.004), but not with age (*r*_s_ = 0.026; *p* = 0.765) or CS glucose (*r*_s_ = 0.025; *p* = 0.802). An inverse correlation was found between CS cortisol and CS insulin levels (*r*_s_ = −0.242, *p* = 0.009) and a trend with GH (*r*_s_ = −0.170; *p* = 0.066).

### GH during CS

A CS GH level <7.5 ng/mL was present in 70 (56.9%) of 123 patients for whom data were available (Table 5). Compared with children with CS GH ≥7.5 ng/mL, these patients were older (median 2.0 [0.6, 3.6] vs. 0 [0, 0.9] years, *p* < 0.001), more commonly born appropriate for gestational age (*p* = 0.039), had higher CS glucose values (42.0 [36.0, 48.0] vs. 35.0 [21.8, 42.5] mg/dL, *p* < 0.001), and were less likely to have CS insulin detected (28.4% vs. 67.3%, *p* < 0.001). Among children with CS GH ≥7.5 ng/mL, hyperinsulinism (either transient or persistent) was diagnosed in 58.5%, compared with 20.0% of those with CS GH <7.5 ng/mL (*p* < 0.01). Hypoglycemia occurred during fasting in 63.8% of patients with CS GH <7.5 ng/mL compared with 20.8% of those with higher GH levels (*p* < 0.001).

**Table 5.** Clinical characteristics of children with spontaneous hypoglycemia stratified by CS GH levels (≥7.5 vs. <7.5 ng/mL)

​	GH <7.5 ng/mL, *n* = 70	GH ≥7.5 ng/mL, *n* = 53	*p* value
Age, median (IQR), years	2.0 (0.6, 3.6)	0 (0, 0.9)	<0.001
Male, *n* (%)	40 (57.1)	33 (62.3)	0.576
Birth weight for gestational age, *n* (%)			
AGA	57 (81.4)	34 (64.2)	0.039
Small for gestational age	5 (7.1)	12 (22.6)	
Large for gestational age	5 (7.1)	4 (7.5)	
Missing	3 (4.3)	3 (5.7)	
Wt-SDS, median (IQR)	−0.87 (−1.93, −0.08)	−1.31 (−2.24, 0.02)	0.524
Ht-SDS, mean±SD	−0.73±1.20	−0.84±1.54	0.733
Clinical presentation, *n* (%)			
Vomiting, sweating, hypothermia	3 (4.3)	1 (1.9)	<0.001
Weakness, syncope, behavioral changes	45 (64.3)	12 (22.6)	
Neonatal hypoglycemia	7 (10.0)	27 (50.9)	
Seizures, tremor, jitteriness	8 (11.4)	11 (20.8)	
Asymptomatic	6 (8.6)	1 (1.9)	
Missing	1 (1.4)	1 (1.9)	
Hypoglycemia timing, *n* (%)			
Fasting	44 (63.8)	11 (20.8)	<0.001
Postprandial	12 (17.4)	12 (22.7)	
Unknown	14 (20.0)	30 (56.6)	
CS glucose, median (IQR), mg/Dl	42.0 (36.0, 48.0)	35.0 (21.8, 42.5)	<0.001
Insulin detected (yes), *n* (%)	19 (28.4)	33 (67.3)	<0.001
Missing	3 (4.3)	4 (7.54)	
CS cortisol, median (IQR), nmol/L	556 (375, 823)	450 (260, 756)	0.148
Diagnosis, *n* (%)			
Unspecified	5 (7.1)	3 (5.7)	<0.01
Persistent HH	12 (17.1)	11 (20.8)	
Transient HH	2 (2.9)	20 (37.7)	
Ketotic hypoglycemia	25 (35.7)	9 (17.0)	
Metabolic disease	9 (12.9)	2 (3.8)	
Hormone deficiency/insufficiency	6 (8.6)	3 (5.7)	
Systemic disease/syndrome	9 (12.9)	5 (9.4)	
Insulinoma	2 (2.9)	0 (0)	
Fasting test peak cortisol, mean±SD, nmol/L	607±215	550±245	0.539
*N*	22	8	
Fasting test peak GH, ng/mL	2.1 (1.5, 3.5)	7.6 (2.9, 16.0)	0.027
*N*	22	8	

CS, critical sample; HH, hyperinsulinemic hypoglycemia; IQR, interquartile range; AGA, appropriate for gestational age.

CS GH levels were associated with CS insulin levels (*r*_s_ = 0.358, *p* < 0.001) and inversely associated with age (*r*_s_ = −0.489, *p* < 0.001) and glucose levels (*r*_s_ = −0.332, *p* < 0.001), but not with Ht-SDS or Wt-SDS (*r*_s_ = −0.046, *p* = 0.694 and *r*_s_ = −0.021, *p* = 0.819, respectively), or CS cortisol (*r*_s_ = −0.17, *p* = 0.066). GH stimulation tests were performed in 28 patients, based on the clinician’s discretion guided by growth patterns and other clinical parameters. In 10 of these patients, a second stimulation test confirmed GH deficiency. Four of the latter also had secondary AI as part of MPHD.

## Discussion

This retrospective study examined cortisol and GH responses to spontaneous hypoglycemia in 133 children presenting over a 30-year period. The principal finding is that while an insufficient cortisol response to hypoglycemia – defined as CS cortisol <500 nmol/L – occurred in approximately half of the patients, AI confirmed by Synacthen stimulation testing was uncommon, affecting only 3.7% of the cohort. A similarly high proportion of patients (56.9%) had CS GH levels below 7.5 ng/mL, yet confirmed GH deficiency was infrequent. These findings challenge the diagnostic utility of isolated CS cortisol and GH measurements in identifying clinically significant hormonal deficiency during spontaneous hypoglycemia in children.

In adults, the frequency of hypoglycemia during adrenal crises has not been extensively studied; one study reported AI in 6.1% of hypoglycemia cases presenting to emergency departments [5, 11]. Our finding of 3.7% in children falls within this range but likely reflects a fundamentally different spectrum of underlying disease. In adults, AI more commonly results from autoimmune adrenalitis, chronic glucocorticoid therapy, or hypothalamic-pituitary pathology [12], whereas in our cohort all 5 patients with confirmed cortisol deficiency had congenital causes – MPHD (*n* = 4) or primary AI as part of Kearns-Sayre syndrome (*n* = 1). Hypoglycemia is a presenting feature in only 6–8% of adults ultimately diagnosed with AI [13], compared with 30–50% of affected children in multiple pediatric series [14, 15], reflecting the greater dependence of younger children on cortisol for glucose homeostasis due to limited glycogen stores and immature gluconeogenic capacity [16].

The high prevalence of low CS cortisol (51.1% <500 nmol/L, 23.3% <276 nmol/L) contrasted with the low rate of confirmed deficiency and is consistent with previous pediatric reports. Thornton et al. [17] report that only 60% of children will have cortisol levels >18 mcg/dL during hypoglycemia. Kelly et al. [9] found that 61% of 151 children undergoing diagnostic fasting had cortisol <18 µg/dL (500 nmol/L), with only 1 patient ultimately diagnosed with central AI. Palladino et al. [18] evaluated 86 children with persistent or severe hypoglycemia and found that 68% had CS cortisol, suggesting an insufficient response, while only 7% had confirmed AI on subsequent testing. Crofton and Midgley [19] demonstrated marked age-dependent differences, with infants under 3 months showing substantially lower median cortisol during spontaneous hypoglycemia (205 nmol/L) compared with older children (>6 months: 1,370 nmol/L), indicating maturation of the cortisol response by approximately 6 months. O’Grady et al. [20] reported that a peak cortisol <500 nmol/L during insulin tolerance testing occurred in 9% of children under 12 years and 25% of those aged 12 or older. Our data extend these observations to a large cohort of children with confirmed spontaneous hypoglycemia and reinforce that CS cortisol values have limited diagnostic specificity in this context.

These age-dependent differences in cortisol responses are physiologically expected and consistent with the immaturity of the hypothalamic-pituitary-adrenal axis in early life. In neonates, attenuated cortisol secretion in response to hypoglycemia may reflect physiologically lower cortisol-binding globulin levels, immature adrenocortical reserve, and blunted central hypothalamic-pituitary-adrenal activation –consistent with this, CS cortisol <500 nmol/L was more prevalent among neonates (60.4%) and infants 1–12 months old (63.6%) in our cohort, than in older children (39.7%).

The threshold of 500 nmol/L (18 µg/dL) has been widely used as the minimum acceptable cortisol response during stress or provocative testing [8, 21]. However, this threshold was derived largely from studies of cortisol responses during ACTH stimulation and insulin tolerance testing – structured diagnostic protocols, not spontaneous hypoglycemic episodes [22]. We applied a uniform 500 nmol/L threshold, consistent with clinical practice, because validated pediatric cutoffs for spontaneous hypoglycemia are lacking. Our finding that 92.6% of children with CS cortisol <500 nmol/L had normal Synacthen responses or cortisol ≥500 on a different sample indicates that this threshold is not appropriate for interpreting values obtained during acute spontaneous hypoglycemia. Given the retrospective design and heterogeneous clinical verification pathways over 30 years, our analyses focused on frequencies and correlations rather than the derivation of diagnostic cutoffs. In the same line, the 276 nmol/L threshold was applied as a secondary, descriptive analysis only and should not be interpreted as a diagnostic cutoff, particularly in neonates, for whom age-specific cortisol thresholds during spontaneous hypoglycemia have not been validated. All 5 patients with confirmed deficiency in our cohort had CS cortisol below 100 nmol/L, yet 6 additional patients with similarly low values had entirely normal Synacthen-stimulated cortisol, underscoring that even a more stringent cutoff has limited positive predictive value. Current guidelines provide no explicit recommendations for interpreting cortisol levels during hypoglycemia. The Pediatric Endocrine Society notes that AI – congenital or acquired – may present with hypoglycemia. If primary AI is suspected, simultaneous measurement of plasma adrenocorticotropic hormone and cortisol is advised, with adrenocorticotropic hormone stimulation testing considered to confirm suspected secondary or tertiary AI [23]. Our data support this approach and emphasize that no CS cortisol value can reliably replace confirmatory dynamic testing. Two patients with hypoglycemia and MPHD initially showed a normal cortisol response to Synacthen stimulation but subsequently developed AI. This highlights the need for close clinical surveillance and repeat testing when primary or central AI is suspected, to prevent missed diagnoses in evolving cases or partial central AI.

The inverse correlation between CS cortisol and CS insulin levels is of particular interest. Children with lower CS cortisol had more frequent insulin detection (53.3% vs. 35.6%, *p* = 0.052) and were more likely to have hyperinsulinism as a final diagnosis. This pattern likely reflects differences in the underlying etiology and mechanism of hypoglycemia rather than true adrenal dysfunction. Hussain et al. [24] demonstrated that neonates with hyperinsulinemic hypoglycemia (HH) frequently generate inappropriately low cortisol responses, with low ACTH levels suggesting central immaturity rather than primary AI. Another proposed mechanism involves insulin-mediated effects on the central nervous system. Given the presence of insulin receptors in the hypothalamus and other neuronal regions, insulin may directly modulate the counter-regulatory hormonal response [25].

Stanley et al. [26] similarly noted that many children with congenital hyperinsulinism had blunted cortisol responses during hypoglycemic episodes despite normal adrenal function. They proposed that the rapid onset and potentially shorter duration of HH may not allow sufficient time for maximal cortisol secretion. Our observation that postprandial hypoglycemia – a pattern suggestive of insulin-mediated mechanisms –was more common in children with lower CS cortisol supports this interpretation.

Children with lower CS cortisol also had significantly higher GH concentrations (7.6 vs. 3.7 ng/mL, *p* < 0.001). This observation may reflect a compensatory counter-regulatory mechanism: in the setting of a blunted cortisol response, greater reliance on GH secretion may be necessary to maintain glucose homeostasis. The association between CS cortisol and both fasting test nadir cortisol (*R* = 0.574) and Synacthen-stimulated cortisol (*R* = 0.448), but not with age or glucose level, provides some reassurance that CS cortisol reflects individual adrenal reserve to a meaningful degree, even if it cannot substitute for formal stimulation testing.

In our cohort, 56.9% of patients had CS GH values <7.5 ng/mL, yet confirmed GH deficiency was infrequent. This mirrors the pattern observed for cortisol and is consistent with previous reports. Kelly et al. [9] reported that 70% of children undergoing diagnostic fasting had GH <7.5 ng/mL, and Hussain et al. [27] documented paradoxically low GH responses during spontaneous hypoglycemia in children despite adequate cortisol responses. The traditional GH cutoff of 7.5 ng/mL was established for provocative testing protocols [28] and may not apply to spontaneous hypoglycemia. Because GH stimulation testing was applied selectively, cohort-wide sensitivity and specificity of CS GH could not be estimated without bias.

We showed that CS GH was inversely associated with age (*r* = −0.489, *p* < 0.001) and positively associated with CS insulin levels (*r* = 0.358, *p* < 0.001). The age-dependent decline is physiologically expected and was reflected in the markedly lower rate of CS GH <7.5 ng/mL in neonates (23.2%) compared with older children (75%, *p* < 0.001). The strong association with insulin detection and the finding that hyperinsulinism was far more prevalent in the group with higher GH values (58.5% vs. 20.0%, *p* < 0.01) suggest that HH, which tends to occur in younger infants and to produce more severe hypoglycemia, provokes a greater GH response. The lack of correlation between CS GH and Ht-SDS or Wt-SDS further supports the interpretation that low CS GH during spontaneous hypoglycemia does not reflect chronic GH deficiency. In keeping with this, isolated GH deficiency rarely presents with hypoglycemia beyond the neonatal period [29]; in our cohort, all 4 patients with confirmed GH deficiency who had secondary AI were diagnosed with MPHD.

The strength of this study lies in the large, real-life cohort of children with true spontaneous hypoglycemia (confirmed glucose <50 mg/dL), using samples obtained as part of routine CS protocols. The long study period and comprehensive endocrine evaluation with extended follow-up enabled reliable identification of AI.

Several limitations deserve mention. The retrospective data collection and reliance on data from children referred to a tertiary endocrine center introduce an inherent risk of selection bias, as the cohort may not reflect the broader population of children presenting with hypoglycemia. Outcome classification was not standardized across the cohort. Furthermore, reliance on Synacthen test and occasional single repeat cortisol may miss evolving or partial central AI. Nonetheless, the prolonged follow-up partially offsets this potential bias. This is relevant, as Shulman et al. [30] reported that a subset of children with borderline adrenal responses may develop overt AI during subsequent follow-up. The absence of a universally accepted cortisol threshold during hypoglycemia in children represents a persistent gap in the literature, and our data cannot resolve this question definitively. Finally, the cortisol assay used in this study differs from the newer immunoassays and liquid chromatography-tandem mass spectrometry methods now in wider use, and assay-specific cutoffs may influence the proportion of patients classified as having insufficient responses [8]. The thresholds applied in this study were established for immunoassay-based measurements and may not translate directly to newer automated immunoassays or LC-MS/MS.

Specifically, the Coat-A-Count RIA overestimates free cortisol relative to LC-MS/MS due to CBG cross-reactivity; applying RIA-derived cutoffs to LC-MS/MS results, or vice versa, may misclassify patients. Future studies using mass spectrometry-based cortisol measurement are needed to reevaluate the diagnostic thresholds applied here.

## Conclusions

Low CS cortisol levels occur in approximately half of children presenting with spontaneous hypoglycemia and, when assessed against Synacthen test results, have limited positive predictive value for confirmed AI. AI, while rare in our cohort (3.7%), is a potentially fatal but entirely treatable condition. A low CS cortisol should therefore prompt confirmatory Synacthen stimulation testing in all cases of unexplained or recurrent pediatric hypoglycemia, irrespective of the specific value obtained.

In neonates, low CS cortisol is particularly frequent (60.4%) and more often reflects immature counter-regulatory physiology or hyperinsulinism than true AI. Nonetheless, in neonates with unexplained or persistent hypoglycemia – especially those with associated features such as micropenis, prolonged jaundice, or midline defects – evaluation for MPHD is warranted.

Low CS GH values are also common (56.9%) and nondiagnostic in isolation. In contrast to AI, isolated GH deficiency is not an immediate threat to life. Further GH evaluation should be guided by growth patterns, auxological data, and clinical judgment rather than by a single CS GH value alone. Formal decision analysis and prospective evaluation are needed to define optimal testing thresholds for cortisol and GH by age and phenotype.

## Statement of Ethics

This study protocol was reviewed and approved by the Institutional Review Board of Rabin Medical Center, Israel, Approval No. RMC-0197-17. The need for informed consent was waived by the Institutional Review Board of Rabin Medical Center, Israel, Approval No. RMC-0197-17.

## Conflict of Interest Statement

Prof. Moshe Phillip was a member of the journal’s Editorial Board at the time of submission. The authors declare no financial or other relationships that might lead to a conflict of interest.

## Funding Sources

No financial assistance was received in support of the study.

## Author Contributions

Merav Gil Margolis: substantial contributions to acquisition of data, analysis and interpretation of data, drafting the article and revising it critically for important intellectual content, and final approval of the version to be published. Michal Yackobovitch-Gavan: substantial contributions to analysis and interpretation of data, revised the article critically for important intellectual content, and final approval of the version to be published. Moshe Phillip: substantial contributions to acquisition of data, revised the article critically for important intellectual content, and final approval of the version to be published. Liat de Vries: substantial contributions to conception and design, acquisition of data, analysis and interpretation of data, drafting the article and revising it critically for important intellectual content, and final approval of the version to be published.

## Data Availability

The datasets generated during and/or analyzed during the current study are not publicly available due to patient confidentiality but are available from the corresponding author on reasonable request.
